# The Rising Trend of the Reverse BBL and the Implications for Aesthetic Surgeons: A Google Trends Analysis

**DOI:** 10.1093/asjof/ojad027.016

**Published:** 2023-04-14

**Authors:** Rachel Safeek, Jonathan Dang, Bruce Mast

**Affiliations:** University of Florida, Gainesville, FL; University of Florida, Gainesville, FL; University of Florida, Gainesville, FL

## Abstract

**Goals/Purpose:**

Aesthetic surgery trends continue to evolve and are often popularized by social media, particularly with celebrity influence, as exemplified by gluteal fat grafting or Brazilian Butt Lift (BBL). There appears to be a trend for reversal of this procedure, but there is a paucity of research describing associated operative techniques and precautions, with most discussion limited to media publications. The aim of this study was to evaluate public interest in reverse BBL surgery over a 5-year period using Google Trends and bring attention to this emerging trend in aesthetic surgery.

**Methods/Technique:**

A Google Trends analysis was completed using the term “reverse BBL”. Trends were analyzed over a 5-year time period and included an assessment of regional searches by state. Changes in search frequency were further analyzed based on media articles describing reverse BBL trends, with the first mention being in August 2021, followed by another series of publications from November-December 2021, and June 2022.

**Results/Complications:**

There was a steady increase in the relative search value (normalized search volume in comparison to total search volume over 5 years) for “reverse BBL” beginning in January 2020, with a peak in December 2021 and June 2022 (Figure 1). The states associated with the most search interest in BBL reversal were: Florida, New York, California, Texas, and Illinois (Figure 2). Average relative search volume significantly increased with media publication of reverse BBL procedures among celebrities, corresponding with August 2021, November- December 2021 and mid-2022 (p<0.05) (Figure 3).

**Conclusion:**

These data indicate there is increasing public interest in reversal of gluteal fat grafting, which appears to correlate with social media trends. As patient’s remorse trends surrounding reverse BBL’s rise, aesthetic surgeons should counsel patients accordingly prior to gluteal augmentation surgery. Furthermore, for patients having undergone gluteal augmentation seeking reversals, pre-operative consultation of risks associated with reverse BBL procedures should be discussed. More research is needed regarding best operative approach for BBL reversal, including approaches with liposuction and additional revision surgeries, and strategies to mitigate postoperative complications, while enhancing cosmetic outcomes.

